# Case Report: genotype–phenotype correlations in *FLNA* mutations: insights from a case of multisystem dysfunction

**DOI:** 10.3389/fgene.2025.1693117

**Published:** 2026-01-05

**Authors:** Jie Liu, Xin Pan, Lina Qiao, Yi Liao, Zhongqiang Liu

**Affiliations:** 1 Department of Pediatric Intensive Care Unit, West China Second Universal Hospital, Sichuan University, Chengdu, China; 2 NHC Key Laboratory of Chronobiology (Sichuan University), Ministry of Education, Chengdu, China; 3 Department of Radiology, West China Second Universal Hospital, Sichuan University, Chengdu, China; 4 Department of Radiology, Sichuan Children’s Hospital, Meishan, China

**Keywords:** congenital short bowel syndrome, filamin A, lung disease, multi-organ involvement, mutation

## Abstract

**Background:**

Filamin A (*FLNA*) mutations are associated with the development of numerous diseases and disorders. Although recent studies have shed light on genotype–phenotype relationships, the evidence remains fragmented.

**Case presentation:**

Herein, we report the case of a male infant with an *FLNA* nonsense mutation (c.5265C>G; p.Tyr1755*) identified through trio whole-exome sequencing. The patient exhibited multisystem dysfunction, including periventricular nodular heterotopia, congenital heart disease (perimembranous ventricular septal defect), congenital short bowel syndrome, lung disease, and fatal sepsis. We analyzed this case along with a systematic review of 62 cases of male patients with *FLNA* mutations to explore genotype–phenotype relationships. Results: Following the American College of Medical Genetics and Genomics and the Association for Molecular Pathology guidelines, the variant was classified as likely pathogenic (PVS1, PM2, and PP3). Segregation analysis confirmed maternal inheritance. Standard genetic testing (karyotype and CGH-array) results were unremarkable.

**Conclusion:**

This case expands the phenotypic spectrum of *FLNA* deficiency, linking a nonsense mutation to a severe clinical course with fatal complications such as necrotizing enterocolitis and sepsis, highlighting the need for vigilant multi-organ monitoring.

## 1 Introduction

Filamin A (*FLNA*), located at Xq28 on the X chromosome, encodes an actin-binding protein essential for cell adhesion, migration, and mechanotransduction. Structurally, *FLNA* spans approximately 48 kb and consists of 48 exons, encoding a large homodimeric protein with key domains such as an N-terminal actin-binding domain and 24 immunoglobulin-like repeats. The C-terminal region facilitates dimerization, forming a flexible V-shaped molecule that cross-links actin filaments into dynamic networks and serves as a scaffold for numerous signaling proteins (Sheen et al., 2005). Pathogenic *FLNA* variants are associated with the development of numerous conditions, including periventricular nodular heterotopia (PNH), congenital heart disease (CHD), and Ehlers-Danlos syndrome-like disorders (Reinstein et al., 2013). However, the genotype–phenotype correlations remain unclear. Gain-of-function mutations can cause syndromic diseases, while loss-of-function mutations lead to non-syndromic diseases (De Wit et al., 2011a). *FLNA*-deficient patients are often overlooked or misdiagnosed, highlighting the need to redefine the phenotypic diversity linked to *FLNA* deficiency (Oda et al., 2016). Herein, we report the case of a full-term male infant with a novel *FLNA* mutation and multisystemic dysfunctions, broadening the known phenotypic spectrum of *FLNA*-related disorders.

## 2 Case presentation

**FIGURE 1:**
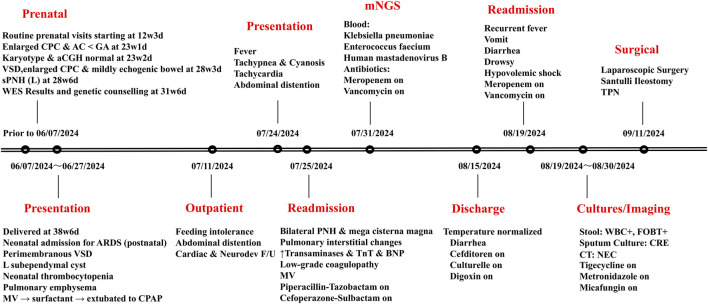
Timeline depicting case progression, cultures/imaging/antibiotic treatment, and mNGS (dates are in the mm/dd/yyyy format). Abbreviations: CPC, cisterna posterior cranial; AC, abdominal circumference; GA, gestational age; sPNH (L), suspected periventricular nodular heterotopia (left); WES, whole-exome sequencing; ARDS, acute respiratory distress syndrome; VSD, ventricular septal defect; MV, mechanical ventilation; CPAP, continuous positive airway pressure; F/U, follow-up; PNH, periventricular nodular heterotopia; TNT, troponin T; BNP, B-type natriuretic peptide; WBC+, pus cells present; FOBT+, fecal occult blood positive; CRE, carbapenem-resistant Enterobacteriaceae; NEC, necrotizing enterocolitis; TPN, total parenteral nutrition.

**FIGURE 2:**
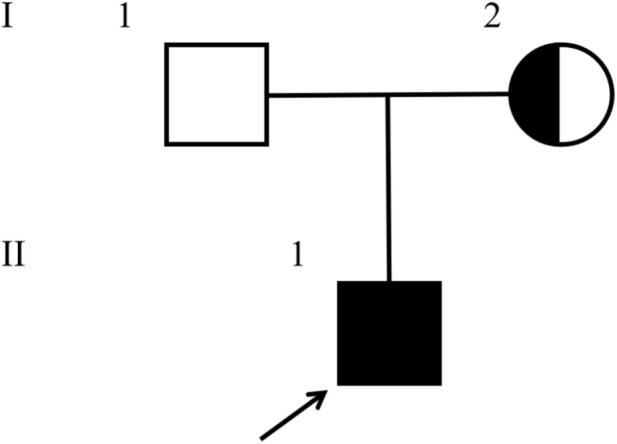
Pedigree of the proband’s family. A heterozygous nonsense mutation (c.5265C>G) in the *FLNA* gene was identified in the proband’s mother (I-2), while the father (I-1) was normal.

**FIGURE 3:**
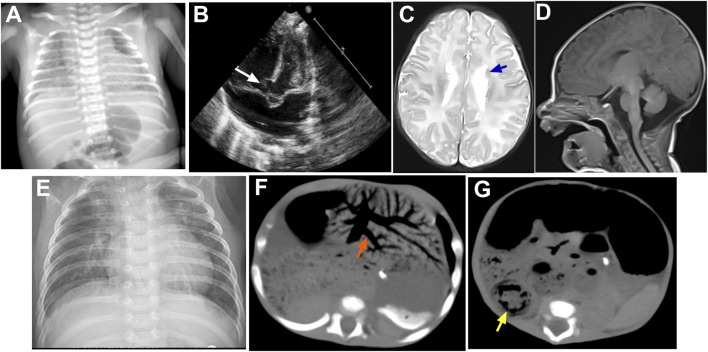
Imaging findings of the patient. **(A)** Chest X-ray demonstrating bilateral diffuse decreased lung lucency; **(B)** transthoracic echocardiography demonstrating perimembranous VSD (white arrow); **(C,D)** cerebral MR imaging demonstrating bilateral PNH (blue arrows) and a large mega cisterna magna; **(E)** chest X-ray showing interstitial changes; **(F,G)** abdominal CT demonstrating extensive hepatic portal venous gas (orange arrow) and intestinal wall thickening (yellow arrow). Abbreviations: VSD, ventricular septal defect; PNH, periventricular nodular heterotopia.

A 2-month-old boy was admitted to the pediatric intensive care unit (PICU) with a fever (39 °C), tachypnea with chest retractions, oxygen saturation of 80% in room air, and tachycardia (180 bpm). The boy showed mild acrocyanosis, diminished subcutaneous fat level (skinfold thickness <3rd percentile), abdominal distention, and a 2–3/6 systolic murmur. The patient had an unusual medical history (Figure 1), but clinical examination revealed no obvious dysmorphism. He was delivered via planned Caesarean section at 38 weeks and 3 days of gestation owing to a fetal ventricular septal defect (VSD) and suspected PNH noted on magnetic resonance imaging (MRI). Prenatal genetic testing was prompted by maternal cardiac ultrasound findings of an asymptomatic ventricular septal aneurysm, paternal abdominal hypopigmented patches, and fetal ultrasound indicating CHD and a widened posterior cranial fossa. A multidisciplinary consultation at 30 weeks of gestation led to trio whole-exome sequencing, which revealed a hemizygous nonsense mutation in *FLNA* (c.5265C>G; p.Y1755*) in the proband. Segregation analysis confirmed that the variant was inherited from his mother (a heterozygous carrier). The proband is the first child of non-consanguineous parents with no family history of *FLNA*-related disorders, which was confirmed using segregation analysis (Figure 2). Karyotype and CGH-array results were normal. At birth, the patient exhibited appropriate anthropometry (weight 2,850 g, height 49 cm, and head circumference 34 cm) and good cardiorespiratory adaptation (Apgar scores 9/10). However, he developed respiratory distress syndrome (Figure 3A) requiring mechanical ventilation, surfactant therapy, and neonatal intensive care unit admission. Subsequent examinations revealed a perimembranous VSD (Figure 3B), a left subependymal cystic lesion, thrombocytopenia (platelet nadir 42 × 10^9^/L), and pulmonary emphysema. After 1 month of hospitalization, the patient was discharged with a weight of 3,010 g. Cardiac and neurodevelopmental follow-ups were suggested owing to the high risk of epilepsy and intellectual disability associated with the *FLNA* mutation. Although neurological examination and developmental assessment results were normal, given the high-risk history of the patient, a brain MRI was performed. The MRI indicated bilateral PNH alongside the mega cisterna magna (Figures 3C,D). Additionally, electroencephalography demonstrated frequent centroparietotemporal spikes and spike–wave discharges (sleep-activated), and the chest X-ray showed pulmonary interstitial changes (Figure 3E). Laboratory tests revealed elevated transaminase levels (peak alanine aminotransferase level, 135 U/L; aspartate aminotransferase level, 314 U/L), myocardial damage (peak troponin level, 109 ng/L; B-type natriuretic peptide level, 754.16 pg/mL), and coagulation dysfunction (low-grade coagulopathy). Further metagenomic next-generation sequencing of peripheral blood revealed the presence of *Klebsiella pneumoniae*, *Enterococcus faecium*, and human mastadenovirus B. Consequently, the patient required mechanical ventilatory support and targeted antimicrobial therapy throughout the 21-day hospitalization period. The patient was readmitted to the PICU within 4 days of discharge owing to the recurrence of fever, along with reduced oral intake, drowsiness with impaired alertness, and decreased urine output (<1 mL/kg/h). Isolation of a carbapenemase-producing *Enterobacter cloacae* complex from a sputum culture (≥10^5^ CFU/mL) demonstrated resistance to meropenem (minimum inhibitory concentration [MIC] >8 mg/L) and imipenem (MIC >4 mg/L), along with recurrent episodes of metabolic acidosis (lowest PH: 7.094) and associated electrolyte derangements (particularly hypokalemia and hyponatremia). Despite administering targeted treatment, the patient subsequently developed vomiting, abdominal distension, and hematochezia, accompanied by diminished bowel sounds on auscultation and a progressive increase in C-reactive protein level (from 34.4 to 79.2 mg/L in 48 h). Abdominal computed tomography revealed extensive pneumatosis coli along with bowel wall edema suggestive of necrotizing enterocolitis (NEC) (Figures 3F,G). Owing to persistent symptoms that were unresponsive to maximal medical therapy, the patient underwent laparoscopic intervention. It revealed a markedly dilated small bowel measuring approximately 90 cm in length and 3 cm in diameter, with gradual luminal narrowing beginning from 60 cm distal to the ligament of Treitz (reduced to a diameter of 2 cm). The caecum was mobile and positioned normally, whereas the colonic luminal diameter remained consistent at 1.5 cm. Biopsy of the ileum was performed along with a Santulli ileostomy. Normal ganglion cells were observed, with areas of mucosal erosion, necrosis, and suppurative inflammation. The prolonged and complicated clinical course required comprehensive support, including parenteral nutrition comprising proteins, glucose, fats, and essential vitamins, following consultations with gastroenterologists and nutritionists. However, the patient ultimately developed fatal sepsis, malignant arrhythmia, and multi-organ failure following multiple unsuccessful attempts at extubation.

## 3 Materials and methods

### 3.1 Ethical consideration and data collection

This study was conducted in accordance with the principles of the Declaration of Helsinki and approved by the Ethics Review Committee of West China Second University Hospital, Sichuan University (Approval Code: KJ-2017-046-4). Clinical data were collected from the medical records of the patient.

### 3.2 Genetic and bioinformatic analyses

Genetic analysis was performed using capture-based next-generation sequencing with the reference transcript NM_001456.4 (*FLNA*). Specifically, trio whole-exome sequencing (WES) was performed on genomic DNA extracted from amniotic fluid (fetus) and peripheral blood (parents). The mean depth of coverage for the exome was >100×, with >98% of the target regions covered at least 20×. The novel status of the *FLNA* c.5265C>G variant, initially identified through trio-WES, was confirmed using Sanger sequencing performed as part of the standard clinical validation procedure. Bioinformatic analysis involved the following filtering steps: (1) the sequenced reads were aligned to the reference genome (GRCh38/hg38); (2) variant calling and annotation were performed using the GATK best practices pipeline and the ANNOVAR software; (3) variants were prioritized based on population frequency (gnomAD allele frequency <0.1%), impact of mutations (nonsense, frameshift, and splice-site) on protein function, and inheritance models (X-linked); and (4) the identified *FLNA* variant was cross-referenced against ClinVar and analyzed using *in silico* prediction tools.

Variant interpretation was performed following the American College of Medical Genetics and Genomics and the Association for Molecular Pathology (ACMG/AMP) guidelines (Richards et al., 2015). The *FLNA* c.5265C>G (p.Tyr1755*) variant was classified as “likely pathogenic” based on the application of evidence codes PVS1 (null variant in a gene where loss-of-function is a known mechanism of disease development), PM2 (absent in controls), and PP3 (deleterious based on *in silico* prediction). Segregation analysis confirmed maternal inheritance.

### 3.3 Literature review and statistical analysis

A systematic literature search was conducted on PubMed, including articles published until May 2025. The search terms included “*FLNA* mutations,” “periventricular nodular heterotopia,” “congenital short bowel syndrome,” “filamin A AND lung disease,” “*FLNA* AND male,” and “*FLNA* AND nonsense mutation.” Studies were included if they reported male patients with pathogenic *FLNA* variants and described associated phenotypes. Reviews and studies without clear genotype–phenotype correlations were excluded. A total of 62 cases from 30 publications were included in the qualitative synthesis, and associations between the various mutation types and clinical phenotypes were evaluated using Fisher’s exact test (applied to contingency tables), a suitable choice for the sample size. Statistical significance was defined as two-tailed *P* < 0.05.

## 4 Discussion

We report the case of a male infant with an *FLNA* nonsense mutation (c.5265C>G; p.Tyr1755*), whose phenotype was characterized by the canonical features of bilateral PNH, a perimembranous VSD, and congenital short bowel syndrome (CSBS). These findings, confirmed using genetic testing, are consistent with those of a loss-of-function variant. Beyond this established spectrum, the clinical course of the patient was complicated by nonspecific but life-threatening sequelae, such as recurrent infections with multidrug-resistant organisms and NEC, leading to fatal sepsis and multi-organ failure, likely driven by the underlying multisystem pathology and potential immune dysfunction.

**FIGURE 4:**
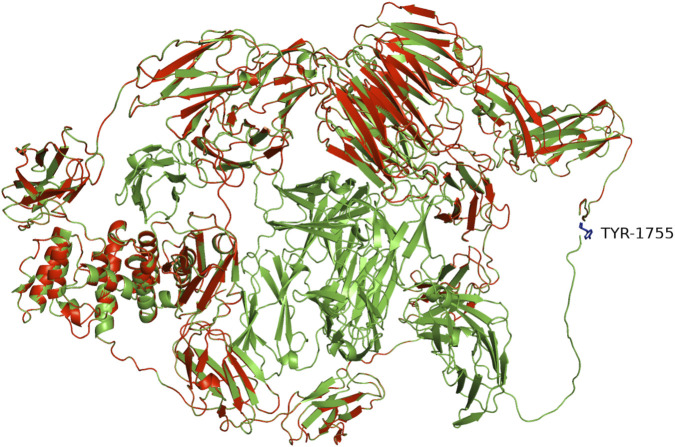
Structural localization of the *FLNA* variant p.Tyr1755*. The schematic illustrates the location of the premature termination codon (p.Tyr1755*) within the domain architecture of *FLNA* (transcript NM_001456.4). This static representation was generated using PyMOL (v3.1, Schrödinger) based on the wild-type *FLNA* structure (AF-P21333-F1, AlphaFold DB); it is intended to visually contextualize the variant. It is not a dynamic functional prediction but aids in hypothesizing potential effects on protein structure. Abbreviations: Tyr, tyrosine.

The core phenotypic triad of PNH, CHD, and CSBS observed in our patient is highly suggestive of *FLNA* haploinsufficiency. The absence of *FLNA* has been reported to cause embryonic lethality and severe malformations in knockout mice (Feng et al., 2006; Stossel, 2010). *FLNA* alterations show diverse phenotypes depending on the molecular mechanism. Truncating mutations are predominantly observed in female patients because they are often lethal in male individuals. Male patients more commonly harbor mosaic, missense, splice-site, or distal truncating variants, aligning with our review of 62 cases of male individuals with *FLNA* mutation (Table 1 summarizes data from the Supplementary Material). Although no genotype–phenotype correlation was evident in the distribution of *FLNA* mutations (Fennell et al., 2015), our analysis of 62 cases yielded two key findings. First, despite a broad phenotypic spectrum, intestinal and skeletal manifestations are the most prevalent and most strongly associated with specific variant types (CNV/frameshift and in-frame deletion/missense, respectively). Second, protein-truncating variants (nonsense, frameshift, and splicing) are indicative of a more severe prognosis, including extensive multi-organ disease and high mortality. These insights are crucial for risk assessment and patient management. Nonsense mutations that introduce premature stop codons and trigger nonsense-mediated mRNA decay, similar to the mutation observed in our case, are often associated with severe cellular dysfunction and worse clinical outcomes compared to most other mutations (Lombardi et al., 2022; Patro et al., 2024). Our patient had a small intestine shorter than that of a full-term infant (190–280 cm) (Siebert, 1980) and had a loss-of-function mutation in *FLNA* (c.5265C>G) in exon 31, resulting in a stop codon at amino acid 1755 (p.Y1755*). Three-dimensional protein modeling demonstrated that the p.Tyr1755* mutation results in a “tailless” protein incapable of dimerization, thereby rendering it nonfunctional and providing a mechanistic basis for the severe clinical presentation (Figure 4). This profound protein dysfunction underlies structural anomalies and may have predisposed our patient to the severe complications observed during the clinical course. Without directly assessing *FLNA* protein levels in affected tissues, we cannot draw causal conclusions. Additionally, nonsense mutations often affect multiple systems, as observed in our patient, leading to complex clinical manifestations that are difficult to treat and result in a poor prognosis. These findings suggest a correlation between nonsense mutations and worse clinical outcomes. Although our analysis is descriptive and definitive conclusions are difficult to draw, the observed patterns may guide future studies with larger samples.

**TABLE 1:** Genotype–phenotype correlations in *FLNA*-related disorders: organ-specific manifestations stratified by variant type among 62 male patients.

Organ system/ Outcome	Synonymous (n = 3)	Missense (n = 18)	In-frame deletion (n = 2)	Nonsense (n = 4)	Frameshift (n = 12)	Splicing (n = 3)	CNV (n = 20)	*P*
Brain	3 (100.0)	8 (44.4)	2 (100.0)	3 (75.0)	7 (58.3)	2 (66.7)	17 (85.0)	0.107
Heart	2 (66.7)	11 (61.1)	1 (50.0)	2 (50.0)	8 (66.7)	3 (100.0)	9 (45.0)	0.675
Lungs	0	8 (44.4)	0	2 (50.0)	5 (41.7)	1 (33.3)	9 (45.0)	0.812
Liver	0	0	0	0	0	0	2 (10.0)	0.571
Kidneys	0	4 (22.2)	2 (100.0)	1 (25.0)	4 (33.3)	1 (33.3)	8 (40.0)	0.371
Intestinal	2 (66.7)	1 (5.5)	1 (50.0)	3 (75.0)	11 (91.7)	2 (66.7)	20 (100.0)	<0.001
Blood	0	1 (5.5)	0	2 (50.0)	1 (8.3)	1 (33.3)	5 (25.0)	0.194
Bones	2 (66.7)	16 (88.8)	2 (100.0)	1 (25.0)	6 (50.0)	2 (66.7)	17 (85.0)	0.032
Skin	1 (33.3)	6 (33.3)	0	0	2 (16.7)	1 (33.3)	2 (10.0)	0.405
Multi-organ^*^	2 (66.7)	6 (33.3)	1 (50.0)	3 (75.0)	6 (50.0)	2 (66.7)	14 (70.0)	0.341
Died	0	4 (22.2)	0	2 (50.0)	5 (41.7)	3 (100.0)	6 (30.0)	0.122

Outcome	Synonymous (n = 3)	Missense (n = 18)	In-frame deletion (n = 2)	Protein-truncating variants (n = 19)			CNV (n = 20)	*P*
Multi-organ^*^	2 (66.7)	6 (33.3)	1 (50.0)	11 (57.8)			14 (70.0)	0.196
Died	0	4 (22.2)	0	10 (52.6)			6 (30.0)	0.190

Abbreviations: CNV, copy number variation.

^*^Multi-organ involvement (>3 organs affected).

The contributions of *FLNA* to intestinal and pulmonary functions have been widely studied (Nhan et al., 2024; Sasaki et al., 2019). *FLNA* is present in cells of the small intestinal muscle layer from early fetal stages, specifically in the smooth muscle cells of the muscularis mucosa and propria. It plays a crucial role in signaling for smooth muscle contractility, essential for normal intestinal function, as demonstrated in animal models (Zada et al., 2023). Although *FLNA* is important for normal small intestinal development (Kapur et al., 2010; van der Werf et al., 2013), its precise role in intestinal development remains unclear (Feng et al., 2006; van der Werf et al., 2015). CSBS is a rare congenital gastrointestinal disease with a poor prognosis in infants, thought to result from interrupted fetal intestinal development owing to limited space in the umbilical cord, vascular issues, or volvulus (Hamilton et al., 1969; Kern et al., 1990). It is also associated with mutations in CXADR-like membrane protein (*CLMP*) and *FLNA*, both crucial for intestinal elongation (Chuang et al., 2020). Seventy cases have been documented to date, with *FLNA* mutations being less common than *CLMP* mutations in fully genotyped patients (Negri et al., 2020; van der Werf et al., 2012; 2013; 2015). The longer form of *FLNA* is essential for normal small intestinal development (Nhan et al., 2024). Some male survivors carrying *FLNA* loss-of-function mutations retain sufficient *FLNA* protein expression to avoid the lethal effects (Oda et al., 2016). *CLMP* mutations mainly affect the intestines, whereas *FLNA* mutations affect multiple systems and are associated with PNH (Robertson et al., 2003). Male patients with CSBS and *FLNA* mutations frequently exhibit multiple congenital anomalies (Van der Werf et al., 2013), as did our patient. Beyond these canonical features, our patient’s clinical trajectory was marked by complications that, while less specific, were critical. The development of refractory NEC and recurrent bloodstream infections likely stems from a confluence of factors. Small intestinal bacterial overgrowth and inflammation are well-established in patients with CSBS (Cole et al., 2010), possibly contributing to refractory NEC in our patient, whereas recurrent sepsis with multidrug-resistant organisms may indicate immune dysfunction. Neutrophils are vital in the early immune response by releasing reactive oxygen species (ROS) and neutrophil extracellular traps (NETs) to combat pathogens. *FLNA* negatively regulates β2 integrin-dependent neutrophil adhesion and ROS production, and its depletion reduces NET release (Uotila et al., 2017). Reportedly, *FLNA* enhances β2 integrin-mediated immunosuppression, while lower *FLNA* levels might hinder transcription factor activation and interleukin-2 production by disrupting PKC translocation, potentially weakening the immune response (Hayashi and Altman, 2006; Uotila et al., 2017). Thus, the recurrent sepsis in our patient may indicate an underlying mutation-related immune deficit. However, the specific role of *FLNA* in immune defense remains unclear, and further assessment was not possible in this study. Further research is needed to elucidate the role of *FLNA* in immune dysregulation, which may facilitate the development of prospective therapeutic strategies.

Childhood interstitial lung disease, caused by genetic, immunological, and developmental lung issues, leads to interstitial lung abnormalities and impaired gas exchange, complicating diagnosis and classification (Kurland et al., 2013; O’Reilly et al., 2015). Most symptoms occur at infancy, in particular, unexplained respiratory distress in full-term neonates (O’Reilly et al., 2015), as observed in our patient. Male patients with respiratory phenotypes associated with rare *FLNA* loss-of-function mutations can survive into infancy owing to residual protein function but often experience a severe course (Desnous et al., 2024). De Wit et al. (2011b) first reported lung disease in patients with *FLNA* mutation. Patients with lung diseases related to *FLNA* mutations have a higher incidence of pneumonia, lung developmental defects, and respiratory failure, which can manifest at infancy (Sasaki et al., 2019). Abnormal *FLNA* interactions affect pulmonary viscoelastic properties and impair alveolar formation and growth (Meliota et al., 2022). Our patient had a slightly lower birth weight for gestational age and pulmonary hypoplasia requiring surfactant replacement therapy. Repeated imaging findings suggested emphysematous changes in the lungs. Additionally, the patient experienced recurrent respiratory failure at 2 months of age, with imaging findings suggestive of interstitial lung abnormalities. Similarly, Kinane et al. (2017) reported multiple unsuccessful extubation attempts. Cardiac color Doppler ultrasound of our patient revealed an increased pulmonary artery forward flow velocity, potentially indicating early pulmonary hypertension (PH), a rare manifestation in carriers of *FLNA* mutations (Hirashiki et al., 2017; Kinane et al., 2017). Pulmonary computed tomography and invasive hemodynamic assessment via catheterization were deferred owing to the high-risk condition of the patient and heightened radiosensitivity of infant tissues, and also based on the as-low-as-reasonably-achievable principle. Nevertheless, his persistent lung disease and hypoxemic respiratory failure, which are recognized in *FLNA*-related disorders, may have contributed to the development of PH and created a vicious cycle of clinical decline. Owing to the poor prognosis of PH, genetic testing is advised for pediatric cases. Moreover, pre-transplant evaluation may be considered, as CHD in *FLNA*-related disorders can complicate PH diagnosis (Demirel et al., 2018).

We discovered a novel loss-of-function mutation in *FLNA* associated with multi-organ involvement in a male patient, particularly atypical respiratory and gastrointestinal symptoms. This finding expands the phenotypic range of *FLNA*-related disorders, suggesting a role for *FLNA* in lung development, intestinal motility, and immune function. However, the lack of assessment of *FLNA* expression in intestinal tissues, quantitative assessment of immune cell functionality, and functional validation of *FLNA*—including measurement of residual protein expression—restricts causal conclusions. Furthermore, morphological features were not documented photographically owing to the patient’s demise, preventing visual validation. Future studies should delve deeper into the potential role of *FLNA* in the involved organ systems and determine whether earlier interventions could have changed the reported outcomes.

In conclusion, this case expands the phenotypic spectrum of a rare *FLNA* nonsense mutation by delineating between well-established, typical manifestations and severe, nonspecific complications that likely represent the downstream effects of multisystem failure and immune dysregulation. This distinction refines the phenotypic map of *FLNA*-related disorders and underscores the importance of vigilant multi-organ surveillance and early aggressive management in affected patients.

## Data Availability

The original contributions presented in the study are included in the article/Supplementary Material, further inquiries can be directed to the corresponding author.
