# Supplementary material for: Case Report: genotype–phenotype correlations in FLNA mutations: insights from a case of multisystem dysfunction
Source: Front Genet. 2026 Jan 5;16:1693117. doi: 10.3389/fgene.2025.1693117 (PMC12812397; doi:10.3389/fgene.2025.1693117)
Supplement: Supplementary file 1 [file Table1.docx]

Supplementary Material: Summary of clinical characteristics of males with FLNA mutations

| Reference | GA/ BBW(g) | Age at  Presentati-on | Neurological | Cardiovascular | Pulmonary | Hepatic | Urinary | Intestinal（Bowel length (cm)） | Skeletal | Skin | Haematological | Additional information | Mutation type | Outcome |
| --- | --- | --- | --- | --- | --- | --- | --- | --- | --- | --- | --- | --- | --- | --- |
| FitzPatrick et al, 1997 case 1(1) | full-mature/3970g | 10 days | PNH | PDA | NA | NA | cryptorchidism | CIIP, malrotation | abnormal facies | NA | chronic thrombocytopenia | esotropia | intragenic partial  duplication of  FLNA | alive at 6 y |
| FitzPatrick et al, 1997 case 2 | full-mature/3827g | 2 weeks | no clinical signs | PDA | NA | NA | hydronephrosis | CIIP | abnormal facies | NA | thrombocytopenia with giant platelets | NA | intragenic partial  duplication of  FLNA | alive |
| FitzPatrick et al, 1997 case 3 | full-mature/3800g | 3 days | NA | PDA, ASD | NA | partly calcified haemangioma of the left lobe of the liver | NA | CIIP, malrotated, short midgut with dilatation of the  small bowel, autopsy ruled out HSCR | no clinical signs | NA | no clinical signs | NA | intragenic partial  duplication of  FLNA | died at  5 wk |
| Guerrini et al, 2004 family 4(2) | pre-mature/NA | at birth | PNH, polymicrogyria | VSD,ASD,PDA, persistent superior vena cava draining to coronary sinus | NA | NA | hypospadias, cryptorchidism, stenosis | CSBS(33),intestinal malrotation | abnormal facies | NA | thrombocytopenia, low-grade coagulopathy | NA | splicing (IVS25 þ 4delAGGAGGTG) | died at  1 wk |
| Hehr et al, 2006(3) | full-mature/3280g | 1 day | PNH, retrocerebellar- cyst | ASD, pulmonary valve prolapse,  dysplastic  tricuspid valve | NA | NA | NA | malrotation, severe constipation | abnormal facies | NA | NA | inguinal hernia | synonymous  c.1923C>T | alive at  46 mo |
| Gerard-Blanluet et al, 2006 twin1(4) | pre-mature/ 870g | 3 month | PNH,mental retardation, seizure | PDA | severe BPD | NA | NA | NA | enlarged  anterior fontanel | NA | NA | NA | missense c.7922C > T  p.Pro2641Leu | alive at 3 y |
| Gerard-Blanluet et al, 2006 twin 2 | pre-mature/ 1025g | 3 month | PNH | PDA | severe BPD | NA | NA | NA | enlarged  anterior fontanel | NA | NA | NA | missense  c.7922C > T  p.Pro2641Leu | died at 8 mo |
| Gargiolu et al, 2007, patient (IV-1)(5) | full-mature/NA | 3 days | abnormal signal white matter,seizures, spastic diplegia | NA | NA | NA | NA | CIIP,intestinal malrotation, pyloric hypertrophy,ileal volvulus | NA | NA | NA | NA | frameshift c.65-66delAC | alive at  18y |
| Gargiolu et al, 2007, patient (IV-5) | full-mature/4200g | 1 day | NA | NA | NA | NA | NA | CIIP,intestinal malrotation, hypertrophic pyloric stenosis | NA | NA | NA | NA | frameshift c.65-66delAC | died at 8 mo |
| Clayton-Smith et al, 2008 patient 1.1(6) | full-mature/3000g | NA | seizures,mental retardation | right-sided cardiac failure | NA | NA | megacystis, bladder distension | CIIP,  autopsy:dilated colon | abnormal facies | NA | NA | early hypotonia, chest infections | duplication including FLNA+ at least 6 other genes | died at 2 y |
| Clayton-Smith et al, 2008 patient 1.2 | full-mature/2600g | NA | mental retardation, presence of enlarged neurons | NA | NA | NA | NA | CIIP, autopsy:distended intestines | abnormal facies | NA | NA | dev delay,  early hypotonia, chest infections | duplication including FLNA+ at least 6 other genes | died at 9 y |
| Clayton-Smith et al, 2008 patient 1.3 | full-mature/2600g | NA | mental retardation, periventricular  Leukomalacia | NA | NA | NA | NA | CIIP | abnormal facies | thin appearing skin | NA | dev delay,  early hypotonia, chest infections,hand stereotypies | duplication including FLNA+ at least 6 other genes | unclear,  survived  to at  least  16 mo |
| Clayton-Smith et al, 2008 patient 2.1 | full-mature/2700g | NA | mental retardation, spasticity, seizures | NA | pneumonia | NA | NA | CIIP | abnormal facies, subluxation of hips | NA | NA | dev delay,  early hypotonia, chest infections, hand stereotypies, dystonic movements | duplication including FLNA+ at least 6 other genes | alive at  10 y |
| Clayton-Smith et al, 2008 patient 2.2 | full-mature/2700g | NA | mental retardation, spasticity, seizures | NA | recurrent  pneumonia | NA | NA | CIIP | bone fractures, abnormal facies | NA | NA | dev delay, chest infections, dystonic movements | duplication including FLNA+ at least 6 other genes | alive at  3 mo |
| Clayton-Smith et al, 2008 patient 2.3 | NA | NA | seizures, mental retardation, spasticity | NA | recurrent  pneumonia | NA | NA | CIIP | bone fractures, abnormal facies | NA | NA | dev delay, chest infections, hand stereotypies, dystonic movements | duplication including FLNA+ at least 6 other genes | alive at  18 y |
| Clayton-Smith et al, 2008 patient 3.1 | post-mature/2830g | NA | partial absence  of corpus  callosum, mental retardation | cardiac failure | recurrent  pneumonia | NA | megacystis, bladder distension, urinary retention | gastroesophageal reflux | abnormal facies | NA | NA | dev delay, early hypotonia, chest infections | duplication including FLNA+ at least 7 other genes | died at 3 y |
| Clayton-Smith et al, 2008 patient 3.2 | full-mature/2900g | NA | partial absence  of corpus  callosum, ventricular dilation, mental retardation | cardiac failure | recurrent  pneumonia | NA | megacystis, bladder distension | CIIP, gastro-oesophageal reflux | abnormal facies | NA | anemia, marrow hypoplasia | dev delay, early hypotonia, chest infections | duplication including FLNA+ at least 7 other genes | died at  10 mo |
| Clayton-Smith et al, 2008 patient 4.1 | full-mature/2700g | NA | seizures, cerebral hypoplasia/  atrophy, mental retardation, periventricular  leukomalacia, spasticity | NA | recurrent  pneumonia | NA | NA | CIIP | contractures, abnormal facies | pale and thin | NA | dev delay, early hypotonia, chest infections, hand stereotypies | duplication including FLNA+ at least 6 other genes | alive at  8 y |
| Clayton-Smith et al, 2008 patient 5.1 | full-mature/3160g | NA | partial agenesis of corpus callosum, cortical atrophy | NA | NA | NA | NA | CIIP, malrotation, gastro-oesophageal reflux | abnormal  facies | NA | NA | hypotonia, poor growth, early hypotonia, chest infections | duplication including FLNA+ at least 6 other genes | alive at  “school  age” |
| Clayton-Smith et al, 2008 patient 5.2 | full-mature/2800g | NA | myoclonic  seizures, mental retardation | NA | NA | NA | NA | CIIP, rectal biopsy ruled out HSCR, gastro-oesophageal reflux | abnormal  facies | NA | NA | hypotonia, poor growth, early hypotonia, chest infections, dysautonomia | duplication including FLNA+ at least 6 other genes | alive at  3 y |
| Clayton-Smith et al, 2008 patient 6.1 | full-mature/1800g | NA | seizures,  hypoplasia of  corpus callosum  and vermis, mental retardation | bicuspid aortic  valve | recurrent respiratory infections | NA | megacystis, bladder distension, cryptorchidism | CIIP, gastro-oesophageal reflux | abnormal  facies | NA | NA | growth  retardation,  hypotonia, dev delay,early hypotonia, chest infections | duplication including FLNA+ at least 6 other genes | alive at  7 y |
| Clayton-Smith et al, 2008 patient 7.1 | full-mature/NA | NA | periventricular  leukomalacia, mental retardation | NA | recurrent respiratory  infections | NA | NA | CIIP, gastro-oesophageal reflux | abnormal  facies | NA | NA | hypotonia, dev delay, retarded  growth, early hypotonia, chest infections | 650kb duplication near, but sparing  FLNA | alive at  6 y |
| Clayton-Smith et al, 2008 patient 9.1 | full-mature/3550g | NA | NA | PDA,ASD, single umbilical artery, heart failure | NA | NA | mild renal  pelviectasis | CIIP, malrotation | NA | NA | thrombocytopenia, anisomegakaryocy-tes | NA | duplication of FLNA | alive at  16 d |
| Clayton-Smith et al, 2008 patient 10.1 | NA | NA | seizures, mental retardation | NA | frequent severe respiratory infections | NA | NA | chronic  diarrhea | abnormal facies, arachnodactyly, joint hyperlaxity,  scoliosis | NA | NA | dev delay,  hypothyroidis-m,chest infections | 4Mb duplication  including FLNA+  at least 8 other genes | alive at  20 y |
| Clayton-Smith et al, 2008 patient 10.2 | NA | NA | mental retardation | NA | NA | NA | NA | CIIP | abnormal facies | NA | NA | chest infections, severe psychomotor delay | 4Mb duplication  including FLNA+  at least 8 other  genes | died at  18 mo |
| Kapur et al, 2010 Patient 1(7) | full-mature/2866g | 3 days | partial ACC, PNH,moyamoya disease, partial agenesis of the corpus callosum | PDA, VSD | NA | cholelithiasis | cryptorchidism, distended bladder | CIIP, CSBS(115^a^ ), intestinal malrotation | NA | NA | anaemia, thrombocytopenia with giant platelets | bifid uvula | duplication first 28 exons | alive at  17 y |
| Kapur et al, 2010 Patient 5 | full-mature/NA | 12 days | PNH, posterior fossa arachnoid cyst, cerebral and spinal cord infarcts associated with  paralysis of his lower extremities | NA | diaphragmat-ichernia | NA | NA | CSBS(68) | abnormal facies, spina bifida occulta | NA | NA | NA | nonsense  c.7021C>T | died at 6 wk |
| Masurel-Paulet et al, 2011(8) | full-mature/3420g | 3 months | PNH,left cerebellar hemisphere hypoplasia, cisterna magna | PDA, mild aortic valve regurgitation, AO root dilatation | progression to severe  lung disease,  pulmonary hypertension, tracheobronchomalacia | NA | bifid right urinary drainage system | NA | NA | NA | macrothrombocyte-s | hypotonia, supraumbilical hernia, developmental delay | nonsense  c.994delG  p.Lys331* | alive at 6 y |
| van der Werf et al, 2013 Family 1(9) | NA | NA | no clinical signs | NA | NA | NA | NA | CSBS(60) | NA | NA | NA | NA | frameshift c.16-17delCT | died |
| van der Werf et al, 2013 Family 2 | NA | NA | no clinical signs | NA | NA | NA | NA | CSBS(228.6^a^) | NA | NA | NA | synovial lipomatosis | frameshift c.16-17delCT | alive at 40 y |
| Oegema et al, 2013 patient 1(10) | full-mature/3200g | 6 years | bilateral PNH, partial agenesis of the corpus callosum, retrocerebellar cyst, seizures | persistent PDA | NA | NA | NA | CSBS(estimated 1/3 of normal), pyloric stenosis, intestinal malrotation | abnormal facies, narrow chest, V shaped sacral dimple, crowded toes | NA | NA | malnourished, inguinal hernia | frameshift c.7941_7942delCT | alive at 6y |
| Oegema et al, 2013 patient 2 | full-mature/3715g | 6 months | PNH, retrocerebellar cyst, delayed motor development, hypotonia | PDA, dysplastic mitral valve | NA | NA | VUR, URTI | CSBS(estimated 1/3 of normal) | abnormal facies, irregular toe implant, sacral dimple | NA | NA | enlarged and dislocated(wandering) spleen, multiple accessory spleens  hypotonia, drooling | frameshift c.7941_7942delCT | alive at 4.5y |
| Oegema et al, 2013 patient 3 | full-mature/2865g | 7 days | hypotonia, PNH, retrocerebellar cyst | ASD | bronchomala-cia | NA | urinary tract infections | CSBS, malrotation | abnormal facies,  sacral dimple | NA | NA | inguinal hernia | frameshift c.7941_7942delCT | alive at 5 mo |
| Reinstein et al, 2013 patient M1(11) | NA | 10 years | PNH, seizures | MVP, ASD | NA | NA | NA | NA | high-arched palate, joint hypermobility | thin,doughy, no skin  hyperextensibility | NA | supraumbilical hernia | missense  c.853C>T (p.Arg285Cys) | alive at 10 y |
| Reinstein et al, 2013 patient M2 | full-mature/NA | at birth | PNH, hypoplasia of  corpus callosum,  syringomyelia | dilatation of ascending and abdominal aorta, dysplastic aortic, mitral and tricuspid valves, tortuous supra-aortic  vessels, EM of aortic wall:  abnormality of elastic fibers | intractable pulmonary  hypertension with heart failure | NA | NA | autopsy: intestinal  malrotation | joint laxity | elastic skin | NA | NA | frameshift  deletion-insertion  c.5498_5504delCACCCACinsAC | died at 2 mo |
| Reinstein et al, 2013 patient M3 | NA | 5 years | paucinodular PNH | mild MVP, thick mitral valve, aortic dilatation | spontaneous pneumothora-x | NA | NA | NA | Joint hypermobility, pectus carinatum, and mild lumbar scoliosis | soft, mildly hyperelastic skin | NA | prior diagnosis of EDS III, normal skin EM, diaphragmatic eventration, umbilical hernia | missense  c.381G>C p.Lys127Asn | alive at 16 y |
| Reinstein et al, 2013 patient M4 | pre-mature/ 2570g | 1 year | PNH | dysplastic mitral and tricuspid valves, cardiac failure | recurrent pulmonary infections | NA | NA | oral feeding difficulties | increased joint laxity, high palate, caudal appendage | cutis laxa | NA | intrauterine growth retardation, inguinal hernia, hypotonic | missense  c.387C>G p.Ile129Met | alive at 1 y |
| Kasper et al, 2013(12) | NA | 57 years | seizures, PNH, megacisterna magna | NA | NA | NA | NA | NA | NA | NA | NA | NA | synonymous  c.5686G>A | alive at 62 y |
| Sankararaman et al, 2013(13) | pre-mature/NA | NA | NA | NA | respiratory failure | NA | cryptorchidism | NA | abnormal facies, osteodysplasia, scoliosis, sacral dimple, articular dysfunction accompanied by finger/toe malformations | NA | NA | thorax narrow, omphalocele, inguinal hernia, bilateral conductive deafness | missense  c.613T>C/p.Cys205Arg  missense  c.5290G>A/  p.Ala1764Thr | died at 3 mo |
| Fennell et al, 2015 patient 3(14) | full-mature/NA | NA | NA | NA | NA | NA | urethral stenosis | NA | developmental dysplasia of the hip, abnormal facies，craniosynostosis, syndactyly, camptodactyly/arthrogryposis | NA | NA | dev delay, feeding aversion | missense  c.3467C>T  p.Pro1156Leu | alive at 2 y |
| Fennell et al, 2015 patient 4 | full-mature/NA | NA | perisylvian polymicrogyria | NA | laryngoma lacia | NA | NA | NA | craniosynostosis, abnormal facies，camptodactyly/arthrogryposis | NA | NA | umbilical hernia, dev delay, fluctuating hearing loss, recurrent otitis media | missense  c.5169T>G  p.Cys1723Trp | alive at 3 y |
| Oda et al, 2016 patient I(15) | full-mature/3040g | NA | NA | severe MR with MVP moderate AR dilatation of the sinus of valsalva, the ascending aorta and the bilateral pulmonary arteries | bilateral pneumothora-x | NA | NA | CIIP, malrotation, crohn’s disease | pectus  excavatum | thin | thrombocytopenia | bilateral inguinal hernias | exon skipping  4-bp deletion in exon 40 | alive at 19 y |
| Oda et al, 2016 patient II | pre-mature/ 2154g | NA | no clinical signs | moderate MR with MVP, ASD | NA | NA | cryptorchidism | CIIP, malrotation | NA | NA | no clinical signs | bilateral inguinal hernias | exon skipping  4-bp deletion in exon 40 | alive at 11 y |
| Atwal et al, 2016 patient 1(16) | post-term/ NA | NA | NA | congenital aortic andmitral valve stenosis | NA | NA | NA | NA | articular dysfunction | spontaneou-s keloid scars, melanocytic nevi, light brown macules | NA | NA | missense  c.4726G>A p.G1576R | alive at 36 y |
| Atwal et al, 2016 patient 2 | NA | NA | NA | pulmonic stenosis, tricuspid regurgitation, aortic stenosis | NA | NA | NA | CIIP | articular dysfunction | spontaneou-s keloid scars | NA | NA | missense  c.4726G>A p.G1576R | alive at 24 y |
| ALYSON et al, 2016(17) | full-mature/NA | NA | PNH, seizures, focal cord atrophy | PDA, aortic valve dysfunction | NA | NA | NA | CSBS, malrotation | joint hyperextensibility | skin laxity | NA | NA | synonymous  c.6769G>C  p. Ala2257Pro | alive at 16 y |
| Carrera-Garcí-a et al, 2017 case 1(18) | NA | NA | PNH, underdevelope-d corpus callosum | mild mitral regurgitation | NA | NA | bilateral cryptorchidism, coronal hypospadias | malrotation | abnormal facies | NA | NA | intranuclear  androgen receptor was negative | deletion  p.2622_2623 deletion | alive at 13y |
| Carrera-Garcí-a et al, 2017 case 2 | NA | NA | PNH, underdevelope-d corpus callosum | NA | NA | NA | bilateral cryptorchidism | NA | abnormal facies | NA | NA | intranuclear  androgen receptor was very low | deletion  p.2622_2623 deletion | alive at 18y |
| Ritelli et al, 2017(19) | NA | NA | NA | severe polyvalvular heart dysplasia, chronic heart failure | NA | NA | NA | NA | mildly progressive scoliosis, abnormal facies, joint hyperextensibility,diffuse spondylosis | soft and doughy skin, skin laxity | NA | NA | splice  c.1829-1G>C p.Phe611_Gly615del | died at 46 y |
| Sasaki et al, 2018(20) | full-mature/3400g | 1 day | PNH | tricuspid valve  dysplasia, aortic  valve incompetent,  thickened mitral  valve, PDA | pulmonary  hypertension, interstitial lung disease,  respiratory failure | NA | NA | CIIP | NA | NA | NA | NA | splice site deletion  c.6670-1delG | died at 4 mo |
| Cannaerts et al, 2018 patient II-2(21) | pre-mature/ 2400g | 2 days | PNH | MVR | NA | NA | cryptorchidism | NA | bilateral hip dislocation, abnormal facies, joint hyperextensibility | skin laxity | NA | dev delay | missense  c.7921C>G, p.Pro2641Ala | alive at 5 y |
| Cannaerts et al, 2018 patient 2 | full-mature/NA | at birth | broader interhemispheri-c fissures and subarachnoid spaces with echogenic parenchyma | MVP, ASD, dilation of the  pulmonary arteries, right atrial and ventricular dilatation | underdevelop-ed left lung，recurrent bronchitis, pulmonary hypertension | NA | hydroureteronephrosis, hypospadias, | chronic diarrhea | hypermobile joints, abnormal facies | translucent skin | NA | bilateral inguinal hernia, muscle hypotonia, food allergies, | frameshift  c.7923delC, p.Tyr2642Thrfs*63 | died at 2 y |
| Yapijakis et al, 2020(22) | NA | NA | NA | NA | NA | NA | NA | NA | cranial hyperostosis, skeletal dysplasia, scoliosis, camptodactyly,  arachnodactyly with flexion deformities, hand muscle atrophy | NA | NA | left progressive mixed hearing  loss, left cholesteatoma, congenital inguinal herni | missense  c.3476A>C | alive at 22 y |
| Thieu et al, 2020(23) | full-mature/NA | at birth | seizures, hemorrhage in bilateral hemispheres, tortuous cranial arteries, focal venous sinus stenosis, widened cranial sutures, plagiocephaly, tethered cord | patent foramen ovale with small shunts, collateral vessels involving the descending aorta | tracheomala-cia | NA | bilateral dysplastic kidneys, bladder wall abnormalities, posterior ureteral valves | NA | skeletal dysplasia, midface hypoplasia, macroglossia, retrognathia, butterfly scapulae, abnormally long bones | NA | platelet dysfunction disorder | atrophic irides bilaterally | missense  p.A1175P | died at 4 mo |
| Qingya Ye et al, 2021(24) | full-mature/3650g | 2 years | NA | NA | NA | NA | NA | NA | abnormal facies, osteodysplasia | NA | NA | NA | missense  c.3527G>A p.Gly1176Glu | alive at 33 mo |
| Dissanayake et al, 2021(25) | full-mature/3200g | 11 years | no clinical sings | no clinical sings | no clinical sings | NA | no clinical sings | no clinical sings | skeletal dysplasia | NA | NA | NA | missense  c.3557C>T  p.Ser1186Leu | alive at 11 y |
| Kalayinia et al, 2021(26) | NA | 2 years | no clinical sings | TOF | NA | NA | NA | NA | NA | NA | NA | NA | missense  c.3415C>T  p.Leu1139Phe | alive at 2 y |
| Wang et al, 2021(27) | full-mature/2750g | 14 days | NA | NA | NA | NA | NA | CSBS(75) | NA | NA | NA | NA | nonsense  c.79G*>*T  p.Glu27* | alive at 34 mo |
| Frenkel et al, 2024(28) | pre-mature/ 2500g | at birth | NA | polyvalvular  dysplasia, biventricular hypertrophy, aortic isthmus hypoplasia | respiratory failure | NA | NA | NA | no clinical signs | NA | NA | NA | missense  c.5180C>T  p.P1727L | died at 12 days |
| Desnous et al, 2024(29) | full-mature/NA | at birth | PNH,corpus callosum dysgenesis, mega cisterna magna | tricuspid valve ballooning with moderate insufficiency and discrete aortic valve dysplasia | PAH, diffuse interstitial lung disease | NA | NA | NA | NA | NA | NA | NA | frameshift  c.4235_4258delinsAGCT  p.Val1412Glufs*26 | died at 4 mo |
| Bilin et al, 2024(30) | NA | 16 years | NA | NA | NA | NA | NA | NA | skeletal dysplasia, abnormal facies | NA | NA | central diabetes insipidus | missense  c.586C>T  p.R196W | alive at 16 y |
| Our patient | full-mature/2850g | 2 months | bilateral PNH, a mega cisterna magna | perimembranous VSD, malignant arrhythmia | ARDS, pulmonary emphysema, pneumonia, respiratory failure, suspected PAH | elevated liver enzymes | no clinical signs | CSBS(90) | no clinical signs | no clinical signs | thrombocytopenia, low-grade coagulopathy | recurrent sepsis with multidrug-resistant organisms | nonsense c.5265C>G,  p Y1755* | died at 4 mo |

Abbreviations: FLNA, Filamin A; GA, gestational age; BBW, birth weight; NA, not available; PNH, periventricular nodular heterotopia; CIIP, Chronic idiopathic intestinal pseudo-obstruction; PDA, patent ductus arteriosus; ASD, atrial septal defect;

HSCR, Hirschsprung's Disease; VSD, ventricular septal defect; CSBS, congenital short bowel syndrome; BPD, Bronchopulmonary Dysplasia; ACC, agenesis of corpus callosum; VUR, Vesicoureteral Reflux; URTI, upper respiratory tract infections;

MVP, mitral valve prolapsed; EM, Electron Microscopy; EDS III, Ehlers-Danlos Syndrome type III; MR, mitral regurgitation; AR, aortic regurgitation; MVR, mitral valve regurgitation; TOF, Tetralogy of Fallot; PAH, Pulmonary Arterial Hypertension;

ARDS, acute respiratory distress syndrome.

^a^In these cases bowel was measured respectively at 10 and 15 years old.

REFERENCES

1. FitzPatrick DR, Strain L, Thomas AE, Barr DG, Todd A, Smith NM, et al. Neurogenic chronic idiopathic intestinal pseudo-obstruction, patent ductus arteriosus, and thrombocytopenia segregating as an X linked recessive disorder. J med genet. 1997;34(8):666-9. DOI: 10.1136/jmg.34.8.666

2. Guerrini R, Mei D, Sisodiya S, Sicca F, Harding B, Takahashi Y, et al. Germline and mosaic mutations of FLN1 in men with periventricular heterotopia. Neurology. 2004;63(1):51-6. DOI: 10.1212/01.wnl.0000132818.84827.4d

3. Hehr U, Hehr A, Uyanik G, Phelan E, Winkler J, Reardon W. A filamin A splice mutation resulting in a syndrome of facial dysmorphism, periventricular nodular heterotopia, and severe constipation reminiscent of cerebro-fronto-facial syndrome. J med genet. 2006;43(6):541-4. DOI: 10.1136/jmg.2005.038505

4. Gérard-Blanluet M, Sheen V, Machinis K, Neal J, Apse K, Danan C, et al. Bilateral periventricular heterotopias in an X-linked dominant transmission in a family with two affected males. Am j med genet a. 2006;140(10):1041-6. DOI: 10.1002/ajmg.a.31197

5. Gargiulo A, Auricchio R, Barone MV, Cotugno G, Reardon W, Milla PJ, et al. Filamin A is mutated in X-linked chronic idiopathic intestinal pseudo-obstruction with central nervous system involvement. Am j hum genet. 2007;80(4):751-8. DOI: 10.1086/513321

6. Clayton-Smith J, Walters S, Hobson E, Burkitt-Wright E, Smith R, Toutain A, et al. Xq28 duplication presenting with intestinal and bladder dysfunction and a distinctive facial appearance. Eur j hum genet. 2009;17(4):434-43. DOI: 10.1038/ejhg.2008.192

7. Kapur RP, Robertson SP, Hannibal MC, Finn LS, Morgan T, van Kogelenberg M, et al. Diffuse abnormal layering of small intestinal smooth muscle is present in patients with FLNA mutations and x-linked intestinal pseudo-obstruction. Am j surg pathol. 2010;34(10):1528-43. DOI: 10.1097/PAS.0b013e3181f0ae47

8. Masurel-Paulet A, Haan E, Thompson EM, Goizet C, Thauvin-Robinet C, Tai A, et al. Lung disease associated with periventricular nodular heterotopia and an FLNA mutation. Eur j med genet. 2011;54(1):25-8. DOI: 10.1016/j.ejmg.2010.09.010

9. van der Werf CS, Sribudiani Y, Verheij JB, Carroll M, O'Loughlin E, Chen CH, et al. Congenital short bowel syndrome as the presenting symptom in male patients with FLNA mutations. Genet med. 2013;15(4):310-3. DOI: 10.1038/gim.2012.123

10. Oegema R, Hulst JM, Theuns-Valks SD, van Unen LM, Schot R, Mancini GM, et al. Novel no-stop FLNA mutation causes multi-organ involvement in males. Am j med genet a. 2013;161a(9):2376-84. DOI: 10.1002/ajmg.a.36109

11. Reinstein E, Frentz S, Morgan T, García-Miñaúr S, Leventer RJ, McGillivray G, et al. Vascular and connective tissue anomalies associated with X-linked periventricular heterotopia due to mutations in Filamin A. Eur j hum genet. 2013;21(5):494-502. DOI: 10.1038/ejhg.2012.209

12. Kasper BS, Kurzbuch K, Chang BS, Pauli E, Hamer HM, Winkler J, et al. Paternal inheritance of classic X-linked bilateral periventricular nodular heterotopia. Am j med genet a. 2013;161a(6):1323-8. DOI: 10.1002/ajmg.a.35917

13. Sankararaman S, Kurepa D, Shen Y, Kakkilaya V, Ursin S, Chen H. Otopalatodigital syndrome type 2 in a male infant: A case report with a novel sequence variation. J pediatr genet. 2013;2(1):33-6. DOI: 10.3233/PGE-13045

14. Fennell N, Foulds N, Johnson DS, Wilson LC, Wyatt M, Robertson SP, et al. Association of mutations in FLNA with craniosynostosis. Eur j hum genet. 2015;23(12):1684-8. DOI: 10.1038/ejhg.2015.31

15. Oda H, Sato T, Kunishima S, Nakagawa K, Izawa K, Hiejima E, et al. Exon skipping causes atypical phenotypes associated with a loss-of-function mutation in FLNA by restoring its protein function. Eur j hum genet. 2016;24(3):408-14. DOI: 10.1038/ejhg.2015.119

16. Atwal PS, Blease S, Braxton A, Graves J, He W, Person R, et al. Novel X-linked syndrome of cardiac valvulopathy, keloid scarring, and reduced joint mobility due to filamin A substitution G1576R. Am j med genet a. 2016;170a(4):891-5. DOI: 10.1002/ajmg.a.37491

17. Hommel AL, Jewett T, Mortenson M, Caress JB. Juvenile muscular atrophy of the distal upper extremities associated with x-linked periventricular heterotopia with features of Ehlers-Danlos syndrome. Muscle nerve. 2016;54(4):794-7. DOI: 10.1002/mus.25175

18. Carrera-García L, Rivas-Crespo MF, Fernández García MS. Androgen receptor dysfunction as a prevalent manifestation in young male carriers of a FLNA gene mutation. Am j med genet a. 2017;173(6):1710-3. DOI: 10.1002/ajmg.a.38230

19. Ritelli M, Morlino S, Giacopuzzi E, Carini G, Cinquina V, Chiarelli N, et al. Ehlers-Danlos syndrome with lethal cardiac valvular dystrophy in males carrying a novel splice mutation in FLNA. Am j med genet a. 2017;173(1):169-76. DOI: 10.1002/ajmg.a.38004

20. Sasaki E, Byrne AT, Phelan E, Cox DW, Reardon W. A review of filamin A mutations and associated interstitial lung disease. Eur j pediatr. 2019;178(2):121-9. DOI: 10.1007/s00431-018-3301-0

21. Cannaerts E, Shukla A, Hasanhodzic M, Alaerts M, Schepers D, Van Laer L, et al. FLNA mutations in surviving males presenting with connective tissue findings: two new case reports and review of the literature. BMC Med Genet. 2018;19(1):140. DOI: 10.1186/s12881-018-0655-0

22. Yapijakis C, Vylliotis A, Angelopoulou A, Adamopoulou M, Chrousos GP, Voumvourakis C. Phenotype and Genotype Study in a Case of Frontometaphyseal Dysplasia 1. Adv exp med biol. 2021;1339(null):319-23. DOI: 10.1007/978-3-030-78787-5_38

23. Thieu T, Milman T, Bhatti TR, Eagle RC. Anterior Segment Dysgenesis With Accessory Iris Membranes in an Infant With Otopalatodigital Spectrum Disorder and Mutation in the FLNA Gene. J pediat ophth strab. 2020;57(null):e8-e11. DOI: 10.3928/01913913-20191230-02

24. Ye Q, Zhao J, Chang G, Wang Y, Ding Y, Li J, et al. Frontometaphyseal dysplasia 1 caused by variant of FLNA gene in a case. Zhonghua Yi Xue Yi Chuan Xue Za Zhi. 2021;38(4):355-8. DOI: 10.3760/cma.j.cn511374-20200523-00374

25. Dissanayake R, Senanayake MP, Fernando J, Robertson SP, Dissanayake VHW, Sirisena ND. Frontometaphyseal dysplasia 1 in a patient from Sri Lanka. Am j med genet a. 2021;185(4):1317-20. DOI: 10.1002/ajmg.a.62058

26. Kalayinia S, Maleki M, Mahdavi M, Mahdieh N. Whole-Exome Sequencing Reveals a Novel Mutation of FLNA Gene in an Iranian Family with Nonsyndromic Tetralogy of Fallot. Labmedicine. 2021;52(6):614-8. DOI: 10.1093/labmed/lmab018

27. Wang Y, Chen S, Yan W, Lu L, Tao Y, Xiao Y, et al. Congenital Short-Bowel Syndrome: Clinical and Genetic Presentation in China. Jpen-parenter enter. 2021;45(5):1009-15. DOI: 10.1002/jpen.1974

28. Frenkel A, Frenkel M, Schulte JJ, Srinivasan S, Lamers L. Polyvalvular Dysplasia and Vascular Abnormalities in a Neonate With an FLNA Variant. JACC Case Rep. 2024;29(18):102556. DOI: 10.1016/j.jaccas.2024.102556

29. Desnous B, Carles G, Riccardi F, Stremler N, Baravalle M, El-Louali F, et al. Diffuse interstitial lung disease in a male fetus with periventricular nodular heterotopia and filamin A mosaic variant. Prenatal diag. 2024;44(3):364-8. DOI: 10.1002/pd.6505

30. Zhang B, Xiang G, Xiang S, Zhang J. Phenotypic and genetic analysis of a Chinese pedigree affected with type 1 Otopalatodigital syndrome. Zhonghua Yi Xue Yi Chuan Xue Za Zhi. 2024;41(5):556-60. DOI: 10.3760/cma.j.cn511374-20230406-00189
